# Rediscovery and redescription of *Centrodora damoni* (Girault) (Hymenoptera: Aphelinidae) from Australia, an egg parasitoid of *Gonipterus* spp (Coleoptera: Curculionidae), after nearly a century

**DOI:** 10.3897/BDJ.4.e7766

**Published:** 2016-04-18

**Authors:** Samantha E Ward, Carlos Valente, Catarina Gonçalves, Andrew Polaszek

**Affiliations:** ‡ Natural History Museum, London, United Kingdom; § RAIZ - Instituto de Investigação da Floresta e Papel, Eixo-Aveiro, Portugal

**Keywords:** egg parasitoid, weevil parasitoid, *
Eucalyptus
*, *
Gonipterus
*, taxonomy

## Abstract

**Background:**

*Centrodora* is a relatively common and widespread genus of morphologically diverse species, and is the most polyphagous genus known within the Aphelinidae, attacking eggs of insects in addition to pupae of Diptera and Hymenoptera, and nymphs of Hemiptera (Polaszek 1991). There are currently about 60 valid species in the genus, but given its morphological and biological diversity, some elevation of species-groups and subgenera to genus-level might be useful in future. *Centrodora* is represented in Australia by twelve species (Noyes 2015).

**New information:**

*Centrodora damoni* (Girault) is redescribed and diagnosed from recently collected specimens reared from the eucalyptus weevil Gonipterus sp. near scutellatus Gyllenhal (Coleoptera: Curculionidae) from Tasmania, Australia. A lectotype is designated from a syntype specimen.

## Introduction

*Centrodora* Foerster is a relatively common and widespread genus of morphologically diverse species, and is the most polyphagous genus known within the Aphelinidae, attacking eggs of insects in addition to pupae of Diptera and Hymenoptera, and nymphs of Hemiptera (Polaszek 1991). There are currently about 60 valid species in the genus, but given its morphological and biological diversity, some elevation of species-groups and subgenera to genus-level might be useful in future. *Centrodora* is represented in Australia by twelve species (Noyes 2015). The genus appears to be of moderate importance as a naturally-occurring primary parasitoid of plant-feeding insects, including some pests (Polaszek 1991). The genus has been split into two species-groups based on the Indian species: the *amoena*-group and *idioceri*-group (Hayat 1998, Hayat 2010). Although *C. damoni* is currently known only from Australia, it fits better within the *idioceri*-group.

*Centrodora damoni* was described by A.A. Girault in 1922 from Queensland as *Aphelinus damoni*, and later transferred correctly to *Centrodora* by Hayat and Fatima 1990. The two syntype females were collected in a forest, and not reared, but later Girault correctly identified a long series, including the first recorded males, from “ova *Gonipterus*” from Canberra.

The host of both the recent and historical material can, unfortunately, only be identified currently as *Gonipterus* sp. Mapondera et al. 2012 have shown that what was known for a long time as a single species *Gonipterus scutellatus* Gyllenhal, commonly known as the eucalyptus snout, beetle eucalyptus weevil or the gum tree weevil, is in fact a complex of at least 10 distinct species, at least 5 of which occur in Tasmania.

## Materials and methods

### Collection

**Figure 1.**
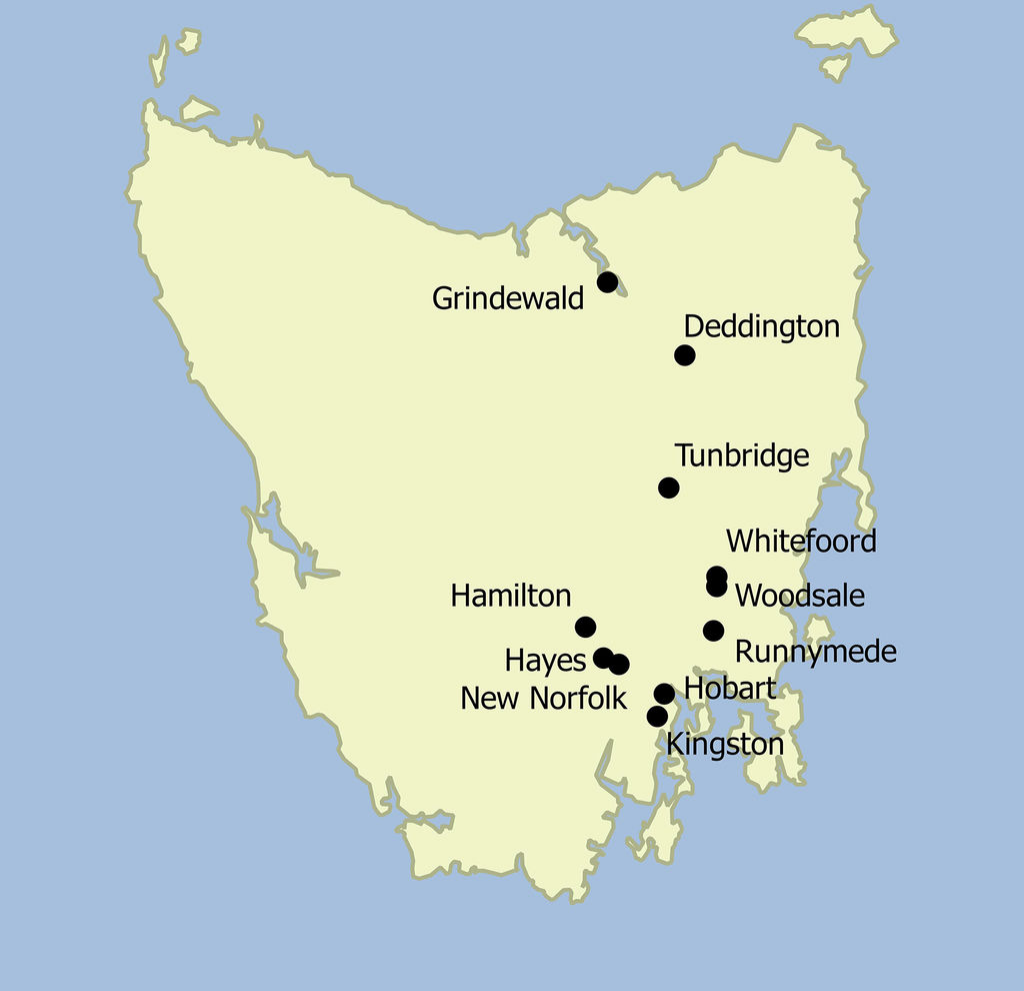
Map of Tasmania indicating sampling sites.

In 2012, field sampling was undertaken on 11 sampling sites in Tasmania, Australia, by the second author (CV). At two of the collection localities, Tunbridge and New Norfolk, *Centrodora damoni* was discovered in egg capsules of *Gonipterus* spp. on *Eucalyptus ovata* and *E. globulus*. At a further three sites (Grindewald, Hamilton and Hayes) the parasitoid emerged from an assortment of egg capsules, see Fig. 1, Table 1.

Specimens were reared from freshly laid *Gonipterus* sp. egg capsules, emerging 54-56 days after parasitism, at 15˚C. The *Centrodora* parasitoids were preserved in 70% ethanol before identification by the fourth author (AP).

### *​* Identification

Specimens preserved in ethanol were extracted for genomic DNA using a “non-destructive” extraction technique (Polaszek et al. 2013). Several PCRs for the 28S D2 and D2-D3 ribosomal DNA fragments using a range of tried and tested primers were unsuccessful. The method of preservation - 70% ethanol, possibly with some methanol present – might have led to degradation of DNA. Attempts will be made in the near future to collect fresh material for DNA sequencing.

Following DNA extraction, specimens were dissected and slide-mounted in Canada balsam following the standard protocol described by Noyes 1982. Photographs were made using a Leitz Ortholux compound microscope with Nomarski Differential Interference Contrast illumination. Images were processed using the stacking software Automontage (Synoptics, Cambridge, UK), and further edited with Adobe Photoshop CC 2014.

### Terminology

Morphological terminology and the format for species descriptions follow Polaszek 1991.

### ​Depositories & Abbreviations

The following institutions provided specimens and/or are depositories for material examined:

BMNH: Natural History Museum, London, UK.

QM: Queensland Museum, Australia.

RAIZ: Instituto de Investigação da Floresta e Papel, Eixo-Aveiro, Portugal.

## Taxon treatments

### Centrodora damoni

(Girault, 1922)

Aphelinus damoni Girault 1922: 208.
Centrodora damoni (Girault) Hayat and Fatima 1990: 250; Dahms 1983: 209.

#### Materials

- **Type status:** Lectotype. **Occurrence:** occurrenceDetails: [AUSTRALIA: Queensland, Wynnum March 1st 1922] Aphelinus damoni Gir. ♀; 3771.; occurrenceRemarks: On a slide with "Coccidoxenus syrphi" (QM). Slide 1 of Dahms (1983).; recordedBy: Girault A.A.; individualCount: 1; sex: female; lifeStage: adult
- **Type status:** Paralectotype. **Occurrence:** occurrenceDetails: [AUSTRALIA: Queensland, Wynnum March 1st 1922] Aphelinus damoni Gir. ♀ (QM).; occurrenceRemarks: Slide 4 of Dahms (1983).; recordedBy: Girault A.A.; individualCount: 1; sex: female; lifeStage: adult
- **Type status:** Other material. **Occurrence:** occurrenceDetails: [AUSTRALIA:] FCT Canberra; ova Gonipterus: Aphelinus damoni Girault [8 ♀♀] (QM).; occurrenceRemarks: Slide 2 of Dahms (1983).; recordedBy: Girault A.A.; individualCount: 8; sex: female; lifeStage: adult
- **Type status:** Other material. **Occurrence:** occurrenceDetails: [AUSTRALIA:] FCT Canberra; ova Gonipterus: Aphelinus damoni Girault [5♀3♂] (QM).; occurrenceRemarks: Slide 3 of Dahms (1983).; recordedBy: Girault A.A.; individualCount: 8; sex: 5 female, 3 male; lifeStage: adult
- **Type status:** Other material. **Occurrence:** occurrenceDetails: 2♀ AUSTRALIA: Tasmania, Nunamara on Gonipterus sp.; C. Valente; DNA1020, 1021 (BMNH/QM); 2♂ AUSTRALIA: Tasmania, multiple sites on Gonipterus sp.; 2012/13; C. Valente; A2, A5 (BMNH/QM).; recordedBy: Valente C.; individualCount: 4; sex: 2 female, 2 male; lifeStage: adult

#### Description

(Figs 2, 3, 4, 5, 6, 7, 8, 9, 10, 11, 12)

*Female*. Body length: 1.00 mm (Fig. 9: Lectotype - length approximate as specimen is dissected and crushed).

**Figure 2.**
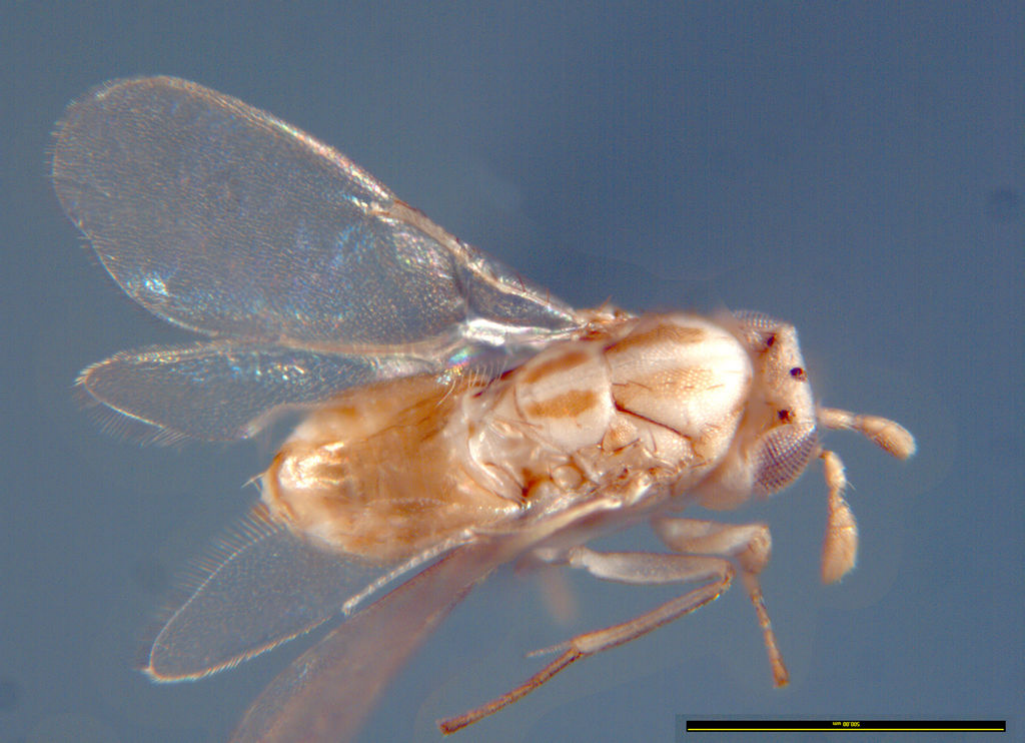
*Centrodora damoni*, female: habitus

*Colour*. Fig. 2. Ground colour cream/off-white. Two broad longitudinal stripes on mesoscutum, extending to scutellum, propodeum laterally, and most of gaster, golden brown. Darker pigmentation on ocelli, notauli, hind tibia and tarsus. Wings hyaline.

##### Morphology

**Figure 3.**
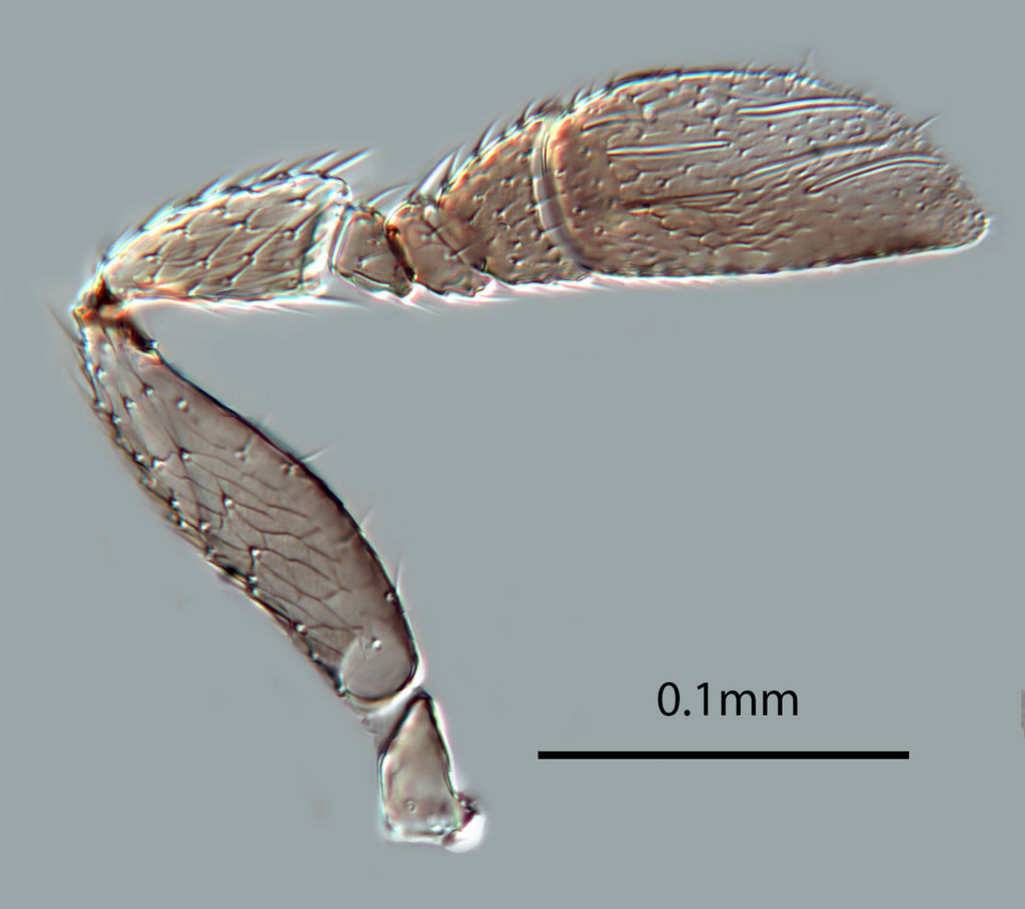
*Centrodora damoni*, female: antenna, outer aspect

**Figure 4.**
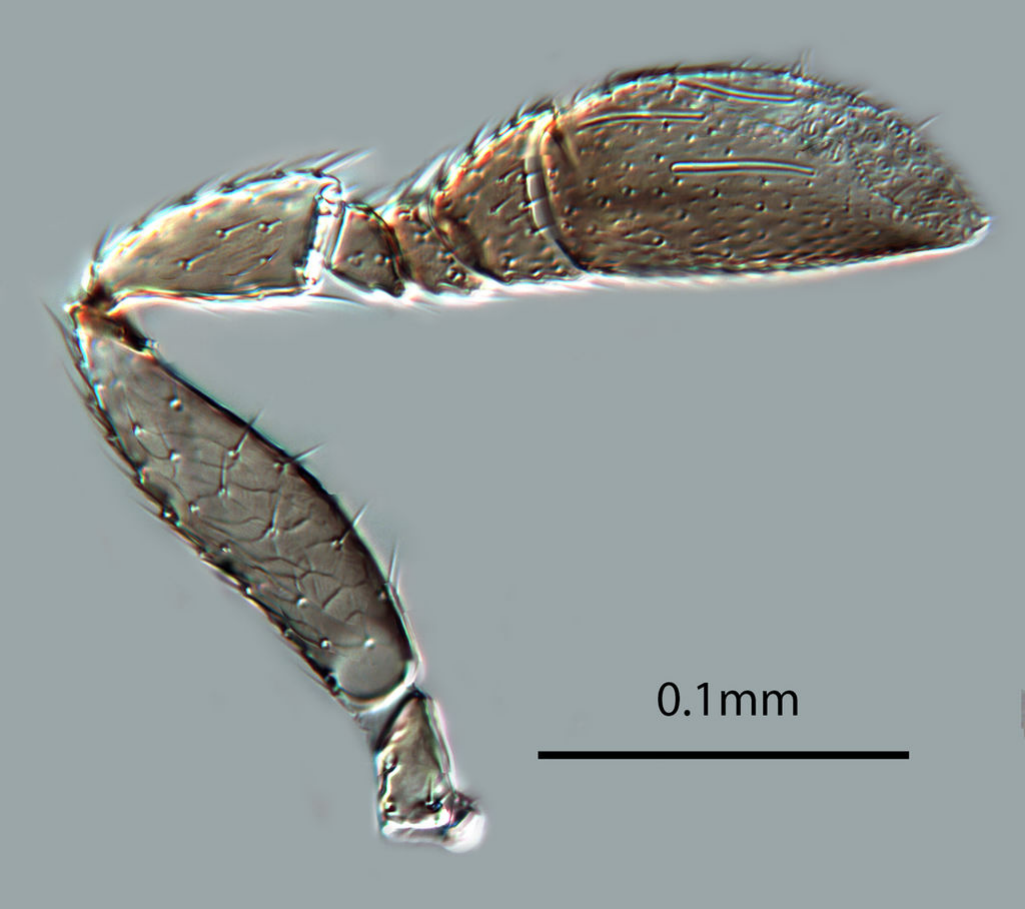
*Centrodora damoni*, female: antenna, inner aspect

*Head*. Frons and antennal scrobes with very fine reticulate sculpture, frons below ocellar triangle with dense robust setae. Maxillary palp two-segmented. Antenna (Figs 3, 4) with radicle 2.1× as long as wide. Scape 4.0× as long as wide, 3.3× as long as radicle, and 2.2× as long as pedicel, flagellum with four ﬂagellom­eres; F1 and F2 combined length longer than F3, F3 1.3× as wide as long, much shorter than pedicel plus F1 and F2, and 0.3× as long as clava; clava with 8-9 multiporous plate sensilla (mps – Fig. 4). Clava with an obliquely truncate apex with numerous basiconic peg sensilla (Fig. 4). Clava 2.1× as long as wide; mps 0.3× length of clava.

**Figure 5.**
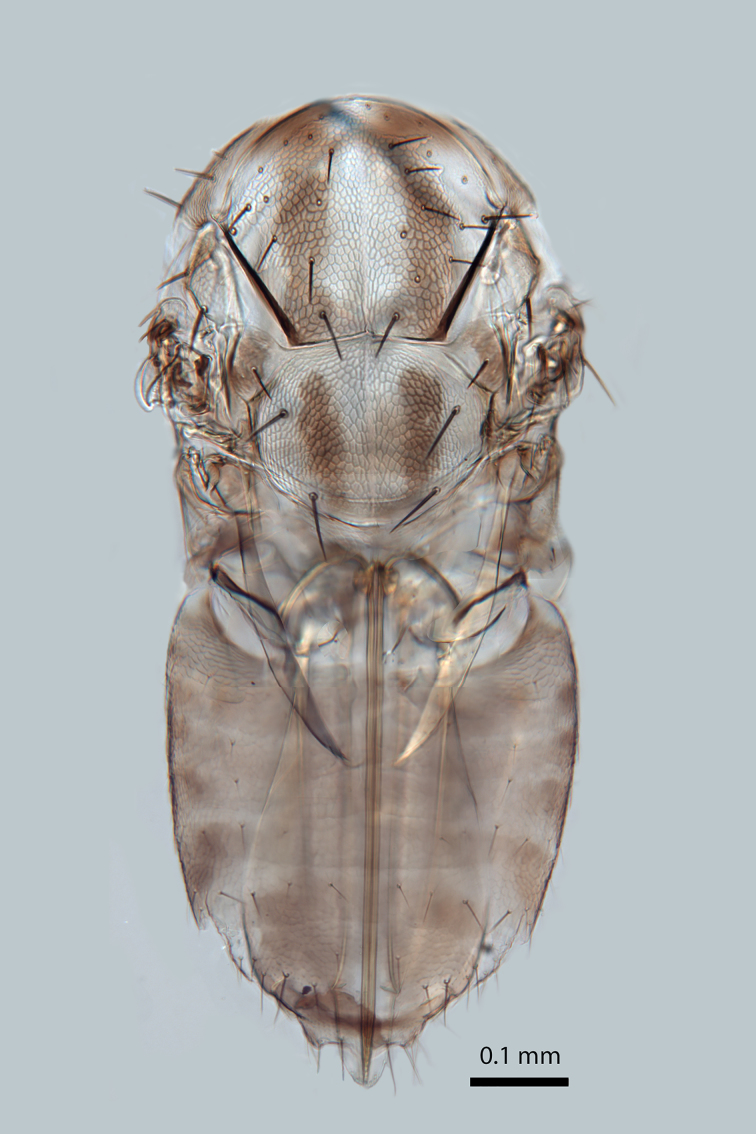
*Centrodora damoni*, female: body in dorsal view

**Figure 6.**
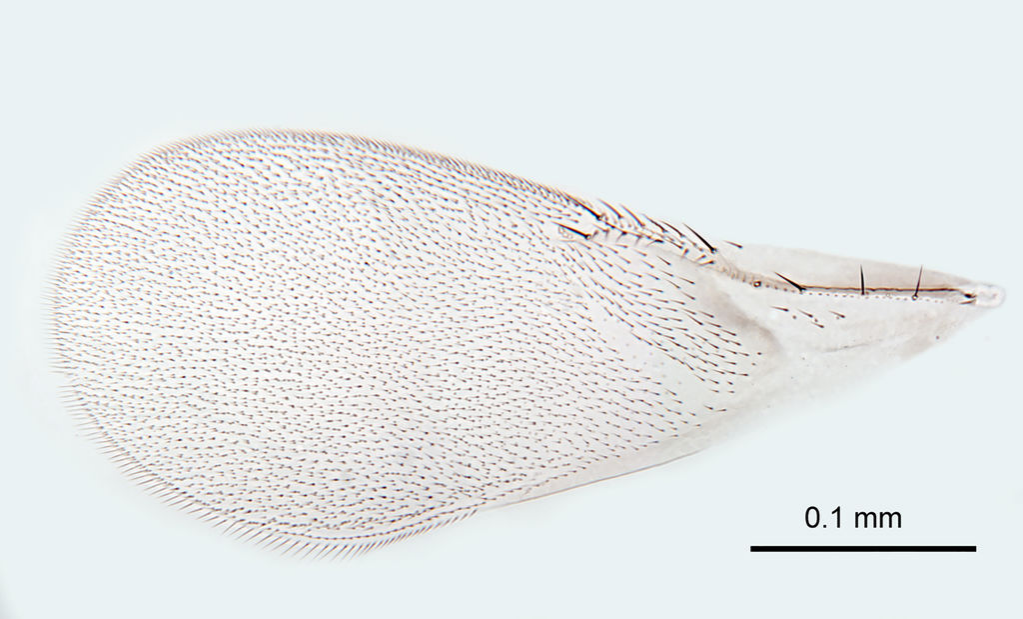
*Centrodora damoni*, female: fore wing

*Mesosoma*. Lateral lobe of mesoscutum with two setae (Fig. 5). Mid lobe of mesoscutum with approximately 13 pairs of setae (Fig. 5) and reticulate sculpture. Scutellum with two pairs of setae (Fig. 5). Fore tibial calcar 0.8× length of basitarsus. Fore wing (Fig. 6) uniformly hyaline, 2.3× as long as broad; longest seta of posterior marginal fringe 0.1× width of wing; marginal vein with row of six long setae along anterior margin; discal setation relatively uniform. Submarginal vein with row of four long setae along anterior margin. Hind wing 3.8× as long as broad, posterior marginal fringe 0.3× width of wing; discal setation relatively uniform.

*Metasoma*. Ovipositor (Fig. 5) 7.7× as long as hind basitarsus. Third valvula approximately 0.2x total ovipositor length.

**Figure 7.**
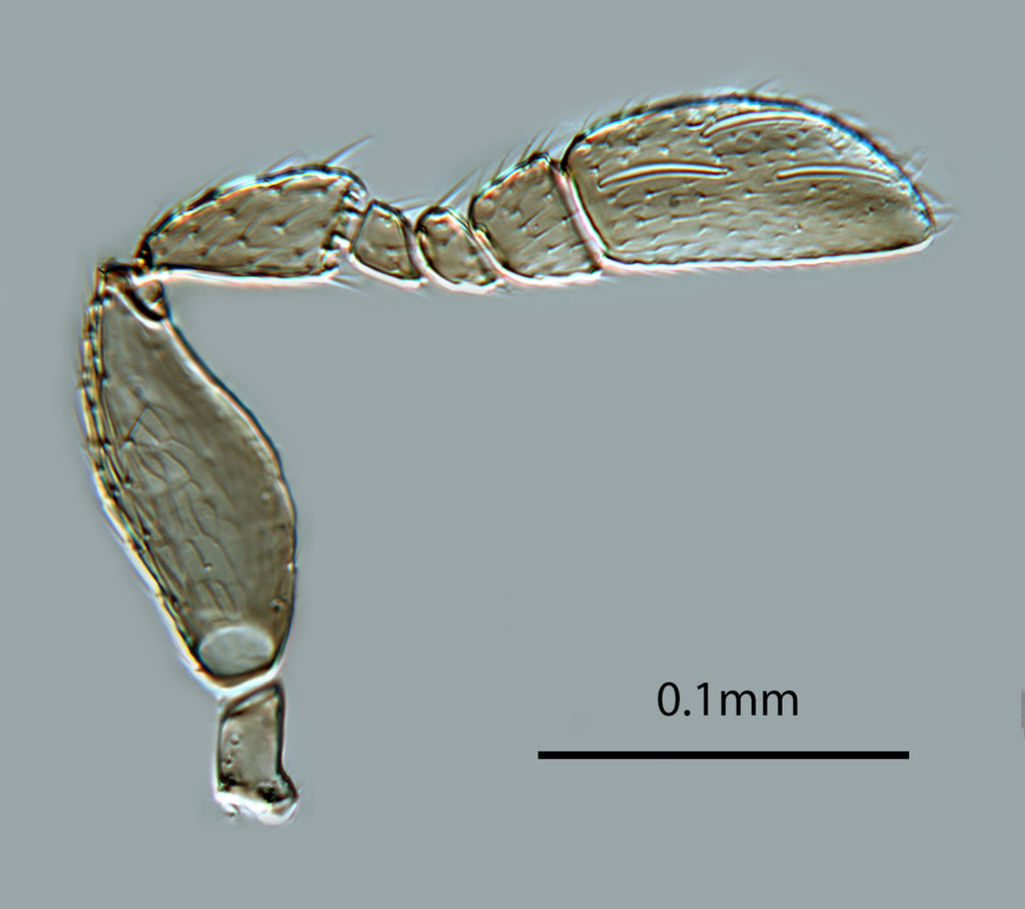
*Centrodora damoni*, male: antenna, outer aspect

**Figure 8.**
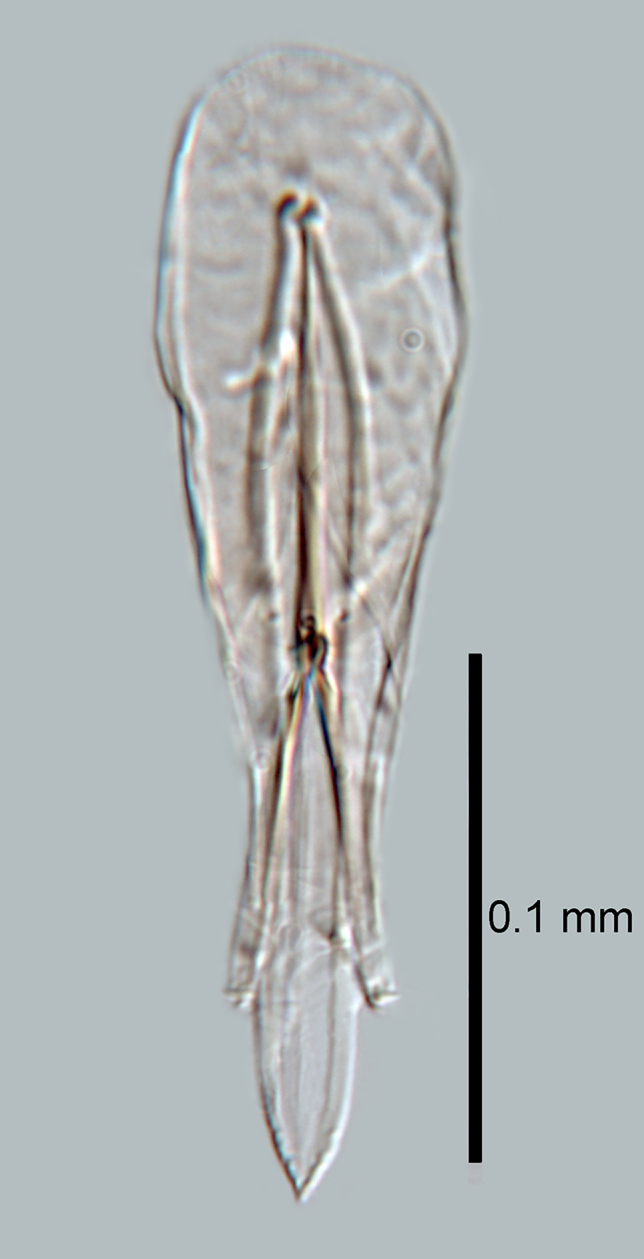
*Centrodora damoni*, male: genitalia

**Figure 9.**
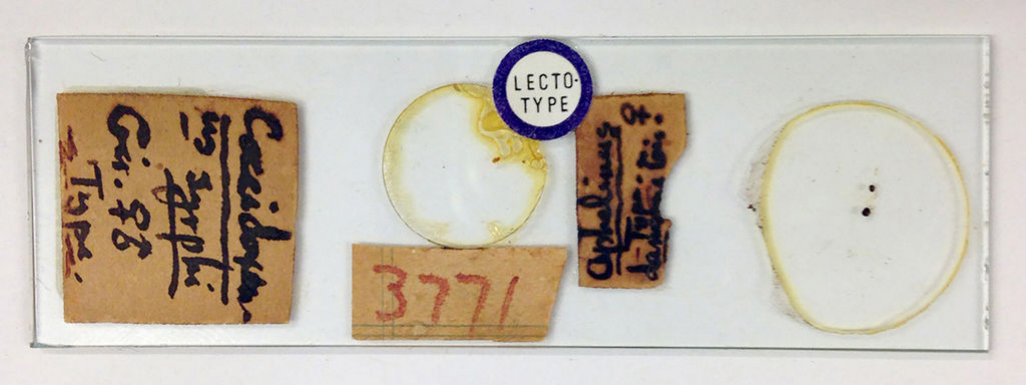
*Centrodora damoni*, lectotype

*Male*. Body length generally 0.8x that of female. Colour and morphology similar to female. Antenna (Fig. 7) with scape shorter and broader than in female (cf Figs 3, 7). Genitalia as in Fig. 8​.

*Host*. *Gonipterus* sp. (Coleoptera: Curculionidae). Based on the type locality of *C. damoni* (Queensland), the host species is likely to be one of the new species mentioned in Mapondera et al. 2012.

#### Diagnosis

*Centrodora damoni* can be distinguished from the other 11 Australian species in the genus by the following combination of characters: Two broad longitudinal stripes on mesoscutum, extending to scutellum; ovipositor less than half total body length (excluding head); apex of antennal clava broadly rounded; fore wing with linea calva present.

#### Distribution

Australia: ACT, Queensland, Tasmania (probably widespread).

## Supplementary Material

XML Treatment for Centrodora damoni

## Figures and Tables

**Figure 10.**
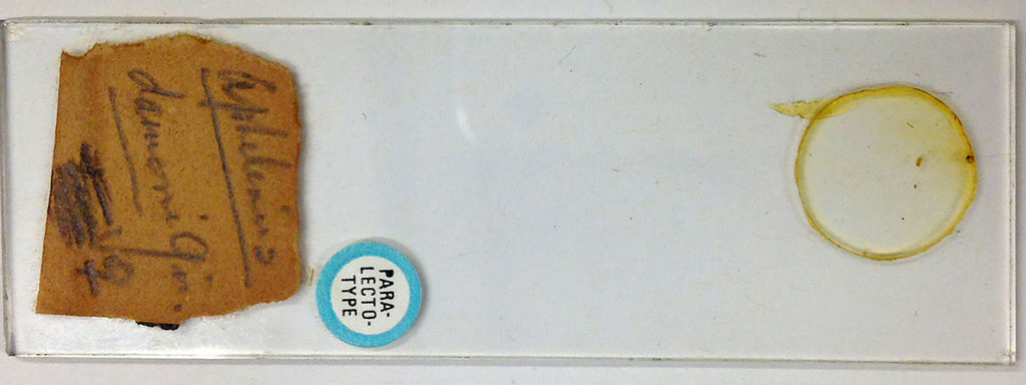
*Centrodora damoni*, paralectotype

**Figure 11.**
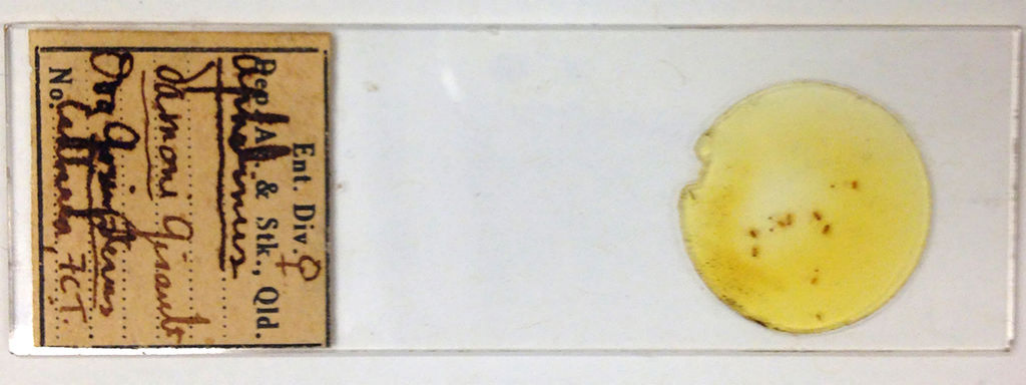
*Centrodora damoni*, other Girault material

**Figure 12.**
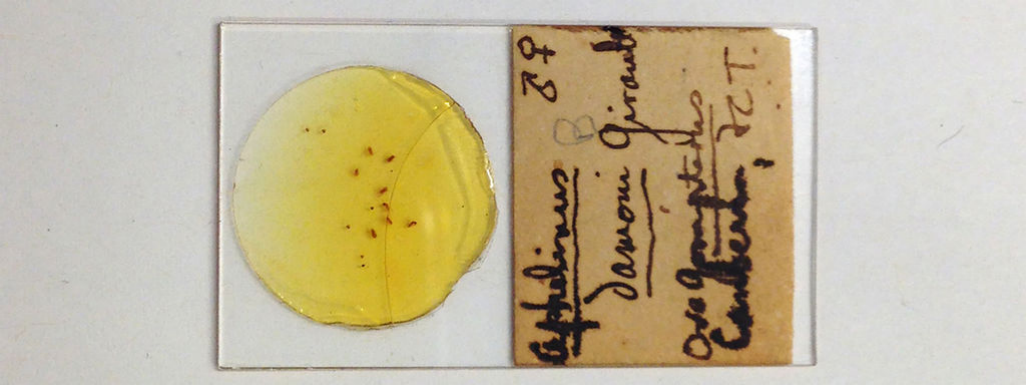
*Centrodora damoni*, other Girault material

**Table 1.** Collection sites at which *Centrodora damoni* was discovered.

**Collection site**	**Latitude**	**Longitude**	**Collection date**	**Egg Capsules collected**	**Nr. of emergences**	**Host**	**Host plant**
Tunbridge	42°07.076	147°19.600	15/21 Nov 2012	1430	6	*Gonipterus* spp.	*Eucalyptus ovata*
New Norfolk	42°47.272	147°03.743	17/23 Nov 2012	90	6	*Gonipterus* spp.	*Eucalyptus globulus*
Grindelwald^1^	41°21.446	147°00.966	19 Nov. 2012	100	14	*Gonipterus* spp.	*Eucalyptus globulus*
Hamilton^1^	42°37.846	146°54.760	23 Nov. 2012			*Gonipterus* spp.	*Eucalyptus ovata*
Hayes^1^	42°45.432	147°00.028	23 Nov. 2012			*Gonipterus* spp.	*Eucalyptus globulus*

^1^ Because few egg capsules were collected at these locations, they were placed on the same container.
